# Validity and reliability of the Italian version of the cardiac quality of life questionnaire for pediatric patients with heart disease (PedsQLTM)

**DOI:** 10.1186/s12872-021-02157-5

**Published:** 2021-08-18

**Authors:** Teresa Grimaldi Capitello, Francesca Bevilacqua, Roberta Vallone, Anna Maria Dall’Oglio, Francesca Santato, Salvatore Giannico, Giulio Calcagni, Simone Piga, Marta Ciofi degli Atti, Simonetta Gentile, Angela Rossi

**Affiliations:** 1 Unit of Clinical Psychology, Department of Neuroscience, Bambino Gesù Children’s Hospital, IRCCS, Rome, Italy; 2 Department of Pediatric Cardiology and Cardiac Surgery, Bambino Gesù Children’s Hospital, IRCCS, Rome, Italy; 3 Unit of Clinical Epidemiology, Medical Direction, Bambino Gesù Children’s Hospital, IRCCS, Rome, Italy

**Keywords:** Health-related quality of life, Pediatric heart diseases, PedsQL cardiac module

## Abstract

**Background:**

Congenital heart disease (CHD) accounts for nearly a third of all major congenital anomalies. Advances in pediatric cardiology shifted attention from mortality to morbidity and health-related quality of life (HRQOL) of patients with CHD and impact on their families. The purposes of this study were to assess the validity and reliability of the Italian version of the Pediatric Quality of Life (PedsQL) Cardiac Module and to create normative data for the Italian population.

**Methods:**

This was an observational cross-sectional study of pediatric patients (aged 2–18 years) with congenital or acquired Heart Disease (HD) and their parents. Families were asked to complete the cardiac pediatric health-related quality of life questionnaire (the Italian PedsQL™ 3.0 Cardiac Module) and the generic pediatric health-related quality of life questionnaire (PedsQL™ 4.0 Generic Core Scales). The sequential validation procedure of the original United States version of the PedsQL™ 3.0 Cardiac Module was carried out under the instruction of the MAPI Research Institute.

To assess construct validity, Pearson’s correlation coefficients were assessed between scores on the Cardiac Module scales and scores on the scales of the General Module. To determine agreement between patient self-report and parent proxy-report, we used intraclass correlation coefficients (ICCs). To evaluate Internal consistency of items, we used Cronbach’s alpha Coefficient.

**Results:**

The study enrolled 400 patients. Construct validity is good between PedsQL Cardiac Module total scores and PedsQL total scores (*p* < 0.001). The recommended standard value of 0.7 was reached on the Cardiac and General Module core scales. Intercorrelations between PedsQL Cardiac module and PedsQL scores revealed medium to large correlations. In general, correlations between Patient self-reports are poorer than Parent-proxy ones.

**Conclusions:**

Cardiac PedsQL scores are valid and reliable for pediatric patients with congenital and acquired HD and may be useful for future research and clinical management.

**Supplementary Information:**

The online version contains supplementary material available at 10.1186/s12872-021-02157-5.

## Background

Congenital heart diseases (CHD) accounts for nearly a third of all major congenital anomalies. The reported incidence of congenital heart disease (CHD) is 7–9 per 1000 live births and remains the leading cause of death for children under 1 year of age [1]. Literature does not provide certain and unambiguous data on the incidence of acquired heart diseases in pediatric population [2]. In recent decades, new surgical techniques and advances in cardiopulmonary bypass, intensive care, heart transplantation, and interventional catheterization have significantly reduced mortality rates for children with heart diseases (HD). Consequently, an increasing number of children are living with chronic HD, 85–90% of whom will reach adulthood, although many of them will require lifelong follow-up [3, 4]. These advances in pediatric cardiology have an influence on morbidity, quality of life (QOL), and health-related quality of life (HRQOL) of children with heart disease, and the impact on their families [4]. Chronic heart disease is often associated with physical symptoms and may impair the patient’s psychological and social well-being and functioning. Therefore, in order to increase overall understanding of the disease, specific tools for assessment of health-related quality of life are advocated [5, 6]. HRQOL is a multidimensional construct which includes physical, psychological and social well-being and functioning [7].

The Pediatric Quality of Life Inventory Version 4.0 Generic Core Scales (PedsQL) is one of the main instruments for measuring pediatric patients’ health related quality of life and one of the very few instruments widely used to assess HRQOL among patients between the ages of 2 and 18 years [8]. PedsQL includes patient and parent versions and assesses different dimensions: physical, emotional, social, and school functioning [9]. Moreover, in order to evaluate HRQoL according to specific clinic conditions, Authors have developed disease-specific modules concerning, for example, ADHD, cancer, asthma and heart disease [10–15]. In particular, Uzark et al. determined reliability and validity of the PedsQL™ 3.0 Cardiac Module as a brief, multidimensional measure of health related quality of life in pediatric cardiology [15]. The instrument has five scales related to symptoms (7 items), perceived physical appearance (3 items), treatment anxiety (4 items), cognitive problems (5 items), and communication (3 items) for parent proxy-report and pediatric patients 8–18 years. The communication scale was not included for toddlers and young children who do not have the cognitive or language ability to verbalize questions and explanations about the heart. An additional treatment barriers scale (5 items) was imbedded in the module to measure adherence issues in subjects taking cardiac medications [15].

The aim of this study was to produce an Italian version of the PedsQL Cardiac Module that is semantically and culturally equivalent to the original English version and to assess its psychometric properties in a sample of pediatric patients with heart disease between 2 and 18 years of age.

## Methods

This was an observational cross-sectional study of pediatric patients with congenital or acquired Heart Disease (HD) and their parents. It was performed at Bambino Gesù Children’s Hospital and Research Institute between September 2016 and December 2017. The study involved patients aged 2–18 with congenital or acquired HD attending an outpatient cardiology unit. Exclusion criteria: major developmental delay that could affect health-related quality of life, developmental problems that limited understanding and/or verbal communication, language barrier. Parents or legal tutors of all patients signed a detailed written informed consent, all patients above 16 years of age signed a written informed assent. All eligible, consenting/assenting patients and parents were enrolled in the study. Participants completed a battery of questionnaires that included: a family form to collect socio-demographic information, the PedsQL™ 3.0 Cardiac Module, and the Italian version of the PedsQL Generic Core Scales 4.0.

As described in previous studies by Uzark et al., do Nascimento et al. and Berkes et al., the PedsQL™ 3.0 Cardiac Module presents a list of items to the patients/parents and asks them to report how problematic each item has been for the patient in the past month [6, 15, 16]. The Cardiac Module provides child self-report and parent proxy-report forms. Ages for child self-report forms are: 5–7, 8–12 and 13–18 years. In parallel, ages for parent proxy-report forms are: 2–4 (toddler), 5–7 (young child), 8–12 (child), and 13–18 (adolescent). In parent-proxy report forms parents are asked to evaluate their child’s HRQOL. Five answer options are listed in the versions for patients 8 to 18 years old and for all versions for parents (0 = it is never a problem; 1 = it is almost never a problem; 2 = it is sometimes a problem; 3 = it is often a problem; and 4 = it is almost always a problem). The answers in the version for children 5–7 years old was simplified to three options (0 = not at all; 2 = sometimes; and 4 = a lot) on a visual scale that exhibited a happy, neutral, and sad face, respectively. As to the scoring, answer options must be transformed as follows: 0 = 100; 1 = 75; 2 = 50; 3 = 25; and 4 = 0. Higher scores indicated a better HRQOL. The sum of all the items over the number of items answered provides mean score. Child self-report and parent proxy-report formats are shown in Additional file 1.

The instruction of the MAPI Research Institute, in accordance with the guidelines of the QOL-SIG TCA (Quality of Life—Special Interest Group Translation and Cultural Adaptation) group, were followed for the validation procedure of the original U. S. version of the PedsQL™ 3.0 Cardiac Module [6, 15–21].

As described in above-cited studies, the validation process begins with two professional native language speakers’ translators who translate the PedsQL™ 3.0 Cardiac Module independently into Italian. Both translators debated the two translated versions with a pediatric cardiologist creating a single agreed version that was translated back into English. This version was sent to the questionnaire’s author for comments and approval. Cognitive interviews with 8 couples of parents of patients with HD aged 2–18 years and with 8 patients aged 5–18 years were performed to control if each item was simple enough to be understood. MAPI Research Institute gave its approval to the so-built final version.

As described by Uzark et al., the PedsQL™ 4.0 Generic Core Scales encompasses Physical Functioning (8 items), Emotional Functioning (5 items), Social Functioning (5 items), and School Functioning (5 items) [15]. The PedsQL™ provides child self-report and parent proxy-report forms. Ages for child self-report forms are: 5–7, 8–12 and 13–18 years. In parallel, ages for parent proxy-report forms are: 2–4, 5–7, 8–12 and 13–18 years. In parent-proxy report forms parents are asked to evaluate their child’s HRQOL. Five answer options are listed in the versions for patients 8 to 18 years old and for all versions for parents (0 = it is never a problem; 1 = it is almost never a problem; 2 = it is sometimes a problem; 3 = it is often a problem; and 4 = it is almost always a problem). The answers in the version for children 5–7 years old was simplified to three options (0 = not at all; 2 = sometimes; and 4 = a lot) on a visual scale that exhibited a happy, neutral, and sad face, respectively. As to the scoring, answer options must be transformed as follows: 0 = 100; 1 = 75; 2 = 50; 3 = 25; and 4 = 0. Higher scores indicated a better HRQOL. The sum of all the items over the number of items answered provides mean score.

### Statistical analysis

We describe the clinical and demographic characteristics of all the patients enrolled in this study. We calculated descriptive statistics and report mean ± SDs or median with IQR and ranges as appropriate for the data distribution. We report percentages for categorical or dichotomous variables.

Acceptability of this study was determined by the low percentage of missing values.

Since the response items were expressed on an ordinal scale, we used Cronbach coefficient to evaluate scale internal consistency reliability [22]. Psychological scales with internal consistency reliabilities of 0.90 are usually recommended for analysing individual patient scores, whereas an internal consistency reliability criterion of 0.70 is recommended for comparing patient groups [23]. Range of measurement was based on the percentage of scores at the minimum and maximum of the scaling range, that is, the minimum possible score (floor effect matches at percentage of scale scores at 0) and the maximum possible score (ceiling effect matches percentage of scale scores at 100).

Study with moderate floor or ceiling effects (> 15%) are considered less precise in measuring latent constructs at the extremes of the scale, similarly study with small floor or ceiling effects (1–15%) are considered to meet acceptable measurement standards [24].

Construct validity was assessed by examining Pearson’s correlation coefficients between scores on the Cardiac Module scales and scores on the scales of the General Module. Pearson Correlation coefficients are defined small if r < 0.30, medium if r ≥ 0.30 and r ≤ 0.50, and large if r ≥ 0.50 [24]. In this regard, we expected large correlations between Cardiac Module total score and Generic Core Scales total score; Heart Problems and Treatment (Cardiac module) and Physical Health (Generic module); Cognitive Problems (Cardiac module) and School Functioning (Generic module).

Since the considered variables can be treated as continuous variables, we used intra-class correlation coefficients (ICCs) to determine agreement between patient self-report and parent proxy-report because it takes into account the ratio between subject variability and total variability [25]. ICCs are defined poor if agreement are less than 0.40, between 0.41 and 0.60 are defined moderate agreement, between 0.61 and 0.80 are good agreement, and between 0.81 and 1.00 are excellent agreement [26].

Statistical significance was assumed as *p* < 0.05 for all tests. All statistical analyses were performed using STATA, Statistical Software: Release 13. College Station, Tx: StataCorp 2013.

## Results

### Subjects’ demographic characteristics

Families of 400 patients were consecutively enrolled in the study. In 318 families, only one parent filled up the questionnaires, in 82 families both parents filled up the questionnaires. Of the 482 parents who took part in the study, 291 (60.4%) were mothers and 191 (39.6%) were fathers. Sociodemographic data of the sample are shown in Table 1.

**Table 1:** Demographic characteristics

Demographic variables	2–4 years		5–7 years		8–12 years		13–18 years		Total	
	N = 37		N = 68		N = 185		N = 110		N = 400	
Children										
Female N(%)	16	43.2	26	38.2	89	48.1	44	40.0	175	43.8

Parents	N = 60		N = 101		N = 197		N = 124		N = 482	
Mothers N(%)	36	60.0	61	60.4	121	61.4	73	58.9	291	60.4
Fathers N(%)	24	40.0	40	39.6	76	38.6	51	41.1	191	39.6
Cohabiting parents N(%)	27	73.0	61	89.7	161	87.1	89	80.9	338	84.5
Single/divorced N(%)	10	27.0	7	10.3	24	12.9	21	19.1	62	15.5
Age M(sd)	37.3	6.2	41.5	5.0	43.4	5.5	48.6	6.0	42.7	5.7
Educational level N(%)										
Compulsory education	7	11.7	15	14.9	38	19.3	17	13.7	77	16.0
High school	30	50.0	46	45.5	92	46.7	69	55.6	237	49.2
Degree	23	38.3	40	39.6	67	34.0	38	30.7	168	34.8
Work N(%)										
Employed	45	75.0	76	75.2	141	71.5	89	71.8	351	72.9
Unemployed	6	10.0	5	5.0	9	4.6	13	10.5	33	6.8
Homemakers	9	15.0	20	19.8	47	23.9	22	17.7	98	20.3

### Subjects’ heart clinical characteristics

Heart diagnosis of the 400 patients and their distribution in age cluster are shown in Table 2. It was not possible to conduct comparisons with a group of healthy children and the cardiologists considered the categories not comparable by disease severity.

**Table 2:** Heart diseases

	2–4 years		5–7 years		8–12 years		13–18 years		Total	
	N = 37		N = 68		N = 185		N = 110		N = 400	
	N	(%)	N	(%)	N	(%)	N	(%)	N	(%)
Aortic anomalies^a^	11	(29.8)	24	(35.3)	67	(36.2)	48	(43.6)	150	(37.5)
Univentricular heart physiology^a^	4	(10.8)	4	(5.9)	15	(8.1)	12	(10.9)	35	(8.8)
Tetralogy of fallot^a^	4	(10.8)	11	(16.2)	33	(17.8)	25	(22.7)	73	(18.2)
RV-PA conduit^a,c^	2	(5.4)	7	(10.3)	13	(7.1)	6	(5.4)	28	(7.0)
Transposition of great arteries^a^	5	(13.5)	9	(13.2)	33	(17.8)	11	(10.0)	58	(14.6)
Cardiomyopathy^b^	1	(2.7)	1	(1.5)	3	(1.6)	4	(3.7)	9	(2.2)
Others	10	(27.0)	12	(17.6)	21	(11.4)	4	(3.7)	47	(11.7)

^a^Congenital heart disease

^b^Acquired heart disease

^c^RV-PA conduit group includes all patients requiring a connection between RV to PA through a conduit instead of the natural RV outflow tract

### Association of PedsQL cardiac module with PedsQL scores

#### Internal consistency-reliability

The internal consistency-reliability values of the 2 modules are presented in Tables 3 and 4, respectively. The recommended standard value of 0.7 was reached on the Cardiac and General Module core scales. Internal consistency was more than 0.7 for some dimensions in the different age groups. Patients in 5–18 age groups and Parents in 2–18 age groups presented high values in Treatment Anxiety scale and Communication scale in Cardiac Module (Table 5).

**Table 3:** Internal consistency reliability of patients PedsQL cardiac module and patients PedsQL generic module (Cronbach coefficient)

Patient	5–7 years			8–18 years		
	All	Male	Female	All	Male	Female
PedsQL cardiac module						
Total SCORE	0.70	0.70	0.79	0.82	0.82	0.85
Heart problems and treatment	0.64	0.55	0.74	0.63	0.68	0.62
Treatment II	0.80	0.80	0.80	0.50	0.61	0.49
Perceived physical appearance	0.67	0.63	0.76	0.71	0.68	0.72
Treatment anxiety	0.94	0.91	0.97	0.88	0.87	0.90
Cognitive problems	0.63	0.62	0.66	0.70	0.60	0.77
Communication	0.88	0.85	0.92	0.74	0.71	0.74
PedsQL generic module						
Total score	0.82	0.77	0.87	0.84	0.82	0.85
Physical functioning	0.72	0.65	0.79	0.73	0.72	0.73
Emotional functioning	0.56	0.51	0.59	0.58	0.52	0.59
Social functioning	0.71	0.62	0.86	0.74	0.59	0.74
School functioning	0.72	0.70	0.77	0.73	0.69	0.77

**Table 4:** Internal consistency reliability of parents PedsQL cardiac module and parents PedsQL generic module (Cronbach coefficient)

	2–4 years	5–7 years	8–18 years
**PedsQL cardiac module**			
Mothers			
Total score	0.57	0.82	0.89
Heart problems and treatment	0.79	0.80	0.76
Treatment II	1.00	1.00	0.56
Perceived physical appearance	0.68	0.49	0.80
Treatment anxiety	0.95	0.94	0.94
Cognitive problems	0.50	0.69	0.84
Communication	1.00	0.76	0.84
Fathers			
Total score	0.84	0.70	0.90
Heart problems and treatment	0.75	0.80	0.82
Treatment II	1.00	0.79	0.78
Perceived physical appearance	1.00	0.55	0.76
Treatment anxiety	0.96	0.95	0.93
Cognitive problems	0.61	0.61	0.84
Communication	0.88	0.78	0.83
**PedsQL generic module**			
Mothers			
Total score	0.91	0.82	0.82
Physical functioning	0.91	0.72	0.72
Emotional functioning	0.74	0.72	0.56
Social functioning	0.86	0.71	0.71
School functioning	0.65	0.72	0.72
Fathers			
Total score	0.85	0.85	0.82
Physical functioning	0.73	0.84	0.72
Emotional functioning	0.79	0.71	0.56
Social functioning	0.86	0.95	0.71
School functioning	0.55	0.77	0.72

**Table 5:** Pearson’s correlation coefficients between cardiac and generic module (n = 400)

	Generic scales				
	Physical health	Emotional functioning	Social functioning	School functioning	Total
Patient version (5–7 years)					
Heart problems and treatment	0.59***	0.135	0.291*	0.203	0.438***
Treatment II	0.166	0.222	0.064	0.014	0.030
Perceived physical appearance	0.307**	0.186	0.300*	0.335**	0.373**
Treatment anxiety	0.167	0.166	0.045	0.074	0.155
Cognitive problems	0.349**	0.325**	0.311**	0.414***	0.458***
Communication	0.373**	0.052	0.400***	0.219	0.354
Total score	0.505***	0.348**	0.397***	0.382	0.545***
Patient version (8–18 years)					
Heart problems and treatment	0.476***	0.380***	0.353***	0.348***	0.458***
Treatment II	0.040	0.021	0.017	0.064	0.001
Perceived physical appearance	0.045	0.121*	0.054	0.096	0.086
Treatment anxiety	0.276***	0.398***	0.210***	0.162**	0.299***
Cognitive problems	0.361***	0.243***	0.274***	0.477***	0.395***
Communication	0.289***	0.232***	0.186**	0.167**	0.259***
Total score	0.392***	0.361***	0.267***	0.291***	0.384***
Parent version (5–7 years)					
Heart problems and treatment	0.922***	0.789***	0.819***	0.767***	0.892***
Treatment II	0.053	0.183	0.084	0.102	0.103
Perceived physical appearance	0.579***	0.596***	0.543***	0.449	0.581
Treatment anxiety	0.752***	0.784***	0.601***	0.647***	0.746***
Cognitive problems	0.697***	0.645***	0.686***	0.739***	0.739***
Communication	0.725***	0.596***	0.649***	0.547***	0.684***
Total score	0.878***	0.835***	0.796***	0.769***	0.880***
Parent version (8–18 years)					
Heart problems and treatment	0.759***	0.713***	0.735***	0.741***	0.773***
Treatment II	0.550***	0.517***	0.538***	0.540***	0.562***
Perceived physical appearance	0.536***	0.567***	0.556***	0.533***	0.570***
Treatment anxiety	0.648***	0.674***	0.627***	0.667***	0.682***
Cognitive problems	0.698***	0.673***	0.701***	0.798***	0.748***
Communication	0.713***	0.669***	0.691***	0.710***	0.729***
Total score	0.730***	0.725***	0.727***	0.753***	0.766***

However, internal consistency was less than 0.7 for some dimensions such as Heart Problems and Treatment scale in Cardiac Module in Patients in 5–18 age groups, in Perceived Physical Appearance scale in Cardiac Module in Children and Parents in 5–7 age groups, and in Cognitive Problems scale in Cardiac Module in Children in 5–7 age groups and Parents in 2–7 age groups.

Internal consistency was more than 0.7 for some dimensions of the Generic Module such as Emotional Functioning in Parents and Patients in 8–18 age groups and School Functioning in Parents in 2–4 age groups in Generic Module.

When considering patients’ gender, some differences were found for internal consistency reliability scores for female versus male patients (Table 3).

##### Comparison of patient and parent in PedsQL Cardiac module and PedsQL scores

Intercorrelations between PedsQL Cardiac module and PedsQL scores revealed medium to large correlations. In general, correlations between Patient self-reports are poorer than Parent-proxy ones.

As hypothesized, PedsQL Cardiac Module total score correlates with PedsQL total score in all age clusters and for both the Patient version and Parent version r > 0.38 and r > 0.88 (*p* < 0.001); Heart Problems and treatment score correlates with Physical Health score r > 0.47 and r > 0.92 (*p* < 0.001); Cognitive problems score (r > 0.41, *p* < 0.001) correlates with School functioning r > 0.79, *p* < 0.001).

##### Parent-patient agreement in cardiac module

The ICCs between Patient self-reports and Parent proxy-reports of the PedsQL™ 3.0 Cardiac Module are given in Table 6. The results obtained were: moderate to good values in both patient and parent groups in the Cardiac Scale; lower values in the Heart problems and treatment, Cognitive problems and Communication Scales across all age groups, and in the Perceived Physical appearance and Treatment anxiety Scales in 8–18 year-olds from the patient group.

**Table 6:** Intraclass correlations (ICCs) between Cardiac Module for child self-report and parent cardiac module proxy-report

Parent–child agreement, ICCs		
PedsQL cardiac module	5–7 years	8–18 years
Heart problems and treatment	0.114	0.216
Treatment II	0.997	0.627
Perceived physical appearance	0.563	0.399
Treatment anxiety	0.411	0.116
Cognitive problems	0.195	0.224
Communication	0.000	0.093
Total score	0.318	0.243

ICCs are designated as 0.40 poor to fair agreement, 0.41–0.60 moderate agreement, 0.61–0.80 good agreement, and 0.81–1.00 excellent agreement

Intraclass Correlations (ICC) showed good agreement in the Perceived Physical appearance and Treatment anxiety Scale scores of children aged 5–7 years and in Treatment II Scale scores of Patients age 8–18 years. ICCs for the Cardiac Module indicated excellent agreement in Treatment II scale in the 5–7 age group.

Additional file 2 shows Reliability and percent Floor and Ceiling Effects for Child Self Report and Parent Proxy-Report for PedsQL™ 3.0 Cardiac Module (Table 7) and PedsQL Generic Core Scales 4.0 (Table 8).

## Discussion

The aim of the present study was to assess psychometric properties of the Italian version of the PedsQL™ 3.0 Cardiac Module and the PedsQL™ 4.0 Generic Core Scale in CHD population. Overall, our results show that the Italian translation of the PedsQL™ 3.0 Cardiac Module is a valid tool to assess HRQoL of Italian pediatric patients with CHD aged 2–18 years.

In particular, internal consistency was generally satisfactory, but nevertheless below the recommended standard value for some dimensions such as Heart Problems and Treatment Scale in patients 5–18 age group, Perceived Physical Appearance in patients and parents in the 2–7 age group and in Cognitive Problems in children and their parents in the 2–7 age group. Similar results had been found in previous studies, Berkes and colleagues in their validation study of the Hungarian Version of PedsQL Cardiac Module ascribe similar results to extremely high frequencies of missing values detected for the Treatment II and Perceived Physical Appearance subscales [6]. In accordance with our results, Gonzales and colleagues, in their validation of the Spanish version, found low internal consistency values in the Perceived Physical Appearance scale, and considered this to be related to the lack of consistency between the items and the neuromaturation and developmental level of the children in the lower age range [27]. Furthermore, when interpreting data of the Cognitive Problems scale, we must remember that in Italy primary school starts from the age of 6, but it frequently happens that children with CHD or other invalidating conditions postpone the beginning of school attending. This might have determined a high percentage of pre-school respondents influencing the data of Cognitive Problems scale. For the same reason, the issue of using school functioning subscale for children aged 2–7 years has been reported as a problem by other European researchers [6, 28, 29].

However, good internal consistency reliability values were found for the Treatment anxiety and Communication Scales, confirming the PedsQL Cardiac Module as a valid measure of the health related quality of life in medical context. This is an interesting finding considering how important it is for clinicians to be aware of how patients experience medical visits and examinations. In fact, as reported by Berkes et al. in accordance with other previous studies, heart diseases had an evident impact on the physical state, but also they considerably influence psychosocial dimensions [6, 7, 15, 16, 30–32].

Interestingly, when considering results of the PedsQL Generic Core Scales 4.0, our data showed low internal consistency in the Emotional Functioning dimension, especially in the 8–18 ages group. This finding seems to show a low emotional awareness in CHD patients in everyday life. As suggested by other Authors, these data may reflect patients’ emotional distress associated with their cardiac condition (see Additional file 2) [7, 33–35].

Lower internal consistency reliability values were calculated in the 2–4 age group for the School Functioning Scales of the Generic Core, which is consistent with previous findings [7]. We must consider that many children in our sample of this age do not attend school because of their disease, and therefore the small sample size could possibly compromise the precision of results.

Intercorrelations between the Cardiac Module Scales and the Generic Scales were significant, in particular the formulated hypothesis (i.e. correlations between total scores; Heart Problems and Treatment vs. Physical Health; Cognitive Problems vs. School Functioning) was confirmed substantiating the construct validity of the tool.

In contrast with previous studies [16], our data showed a poor agreement between child self- and parent proxy-reports, in particular for 8–18 age groups. We can speculate, as reported in literature, a tendency to parental underestimation of QOL and coping mechanisms of chronically ill children which can lead to disagreement [7, 29, 33, 36, 37]. Health care providers should be aware of the frequent disagreement in HRQoL perception which may result in pediatric patients being left alone with their unrecognized emotional distress or, on the other hand, lead to overprotection by parents due to underestimation of their child’s coping skills.

## Conclusions

The Italian version of PedsQL™ 3.0 Cardiac Module is valid and reliable for pediatric patients with congenital and acquired HD and may be useful for future research and clinical management.

## Supplementary Information

Additional file 1: PedsQL™ 3.0 Cardiac Module. The additional file includes all formats of Child Self-Report and Parent Proxy-Report of PedsQL™ 3.0 Cardiac Module.

Additional file 2: Table 7: PedsQL 3.0 Cardiac Module Scores, Reliability and percent Floor and Ceiling Effects for Child Self-Report and Parent Proxy-Report. Table 8: PedsQL 4.0 Generic Module Scores, Reliability and percent Floor and Ceiling Effects for Child Self-Report and Parent Proxy-Report. Tables 7 and 8 show number of items, number of subjects, Mean, SD, percent Floor and Ceiling effects for PedsQL 3.0 Cardiac Module Scores and PedsQL 4.0 Generic Module Scores, respectively.

## Data Availability

This manuscript includes an ‘Availability of data and materials’ statement. Data are stored in Hospital database (and available from the corresponding author on reasonable request).

## Abbreviations

ADHD: Attention deficit and hyperactivity disorder
CHD: Congenital heart disease
HD: Heart disease
HRQOL: Health-related quality of life
ICCs: Intraclass correlations
IQR: InterQuartile range
PedsQL: Pediatric quality of life
QOL: Quality of life
QOL-SIG TCA: Quality of life-special interest group translation and cultural adaptation
SD: Standard deviation
