# Supplementary material for: Validity and reliability of the Italian version of the cardiac quality of life questionnaire for pediatric patients with heart disease (PedsQLTM)
Source: BMC Cardiovasc Disord. 2021 Aug 18;21:398. doi: 10.1186/s12872-021-02157-5 (PMC8371780; doi:10.1186/s12872-021-02157-5)
Supplement: Supplementary file 2 — Additional file 2: Table 7: PedsQL 3.0 Cardiac Module Scores, Reliability and percent Floor and Ceiling Effects for Child Self-Report and Parent Proxy-Report. Table 8: PedsQL 4.0 Generic Module Scores, Reliability and percent Floor and Ceiling Effects for Child Self-Report and Parent Proxy-Report. Tables 7 and 8 show number of items, number of subjects, Mean, SD, percent Floor and Ceiling effects for PedsQL 3.0 Cardiac Module Scores and PedsQL 4.0 Generic Module Scores, respectively. [file 12872_2021_2157_MOESM2_ESM.docx]

| **Cardiac Module Scales** | **Number of items** | **n** | **Mean** | **SD** | **% Floor** | **% Ceiling** |
| --- | --- | --- | --- | --- | --- | --- |
| **Child Self-Report (5-7 years)** |  |  |  |  |  |  |
| Total Score | 25 | 68 | 81.1 | 13.3 | 21.2 | 40.0 |
| Heart problems and treatment | 7 | 68 | 80.0 | 15.8 | 11.7 | 46.9 |
| Treatment II | 3 | 68 | 96.4 | 9.6 | 0.0 | 87.5 |
| Perceived Physical appearance | 3 | 68 | 80.6 | 24.7 | 11.1 | 62.9 |
| Treatment anxiety | 4 | 68 | 79.6 | 30.5 | 10.3 | 61.0 |
| Cognitive problems | 5 | 68 | 85.8 | 16.1 | 3.5 | 57.6 |
| Communication | 3 | 68 | 75.9 | 34.8 | 16.8 | 61.8 |
| **Child Self-Report (8-12;13-18 years)** |  |  |  |  |  |  |
| Total Score | 27 | 295 | 78.8 | 12.5 | 9.6 | 26.6 |
| Heart problems and treatment | 7 | 295 | 78.2 | 13.5 | 3.7 | 34.7 |
| Treatment II | 5 | 295 | 90.8 | 10.3 | 1.6 | 53.8 |
| Perceived Physical appearance | 3 | 295 | 78.9 | 24.8 | 7.3 | 48.9 |
| Treatment anxiety | 4 | 295 | 78.1 | 24.0 | 5.7 | 42.6 |
| Cognitive problems | 5 | 295 | 76.3 | 19.4 | 4.7 | 35.2 |
| Communication | 3 | 295 | 77.7 | 25.1 | 7.8 | 44.7 |
| **Parent Proxy-Report, Father (2-4years)** |  |  |  |  |  |  |
| Total Score | 23 | 37 | 77.6 | 12.1 | 15.6 | 26.7 |
| Heart problems and treatment | 7 | 37 | 80.3 | 16.6 | 7.6 | 36.0 |
| Treatment II | 3 | 37 | 93.3 | 14.9 | 0.0 | 83.3 |
| Perceived physical appearance | 3 | 37 | 98.3 | 7.4 | 0.0 | 90.5 |
| Treatment anxiety | 4 | 37 | 66.9 | 32.9 | 10.8 | 29.7 |
| Cognitive problems | 5 | 37 | 59.1 | 25.0 | 18.2 | 29.6 |
| Communication | 3 | 37 | 82.6 | 26.5 | 5.6 | 50.0 |
| **Parent Proxy-Report, Mother (2-4years)** |  |  |  |  |  |  |
| Total Score | 23 | 37 | 70.6 | 14.3 | 17.1 | 25.0 |
| Heart problems and treatment | 7 | 37 | 71.4 | 21.7 | 10.8 | 33.3 |
| Treatment II | 3 | 37 | 98.1 | 5.6 | 0.0 | 90.0 |
| Perceived physical appearance | 3 | 37 | 91.7 | 14.7 | 0.0 | 67.6 |
| Treatment anxiety | 4 | 37 | 57.6 | 34.5 | 13.3 | 26.7 |
| Cognitive problems | 5 | 37 | 54.2 | 26.5 | 22.1 | 28.6 |
| Communication | 3 | 37 | 78.1 | 27.1 | 7.0 | 45.6 |
| **Parent Proxy-Report, Father (5-7 years)** |  |  |  |  |  |  |
| Total Score | 25 | 68 | 75.4 | 13.2 | 12.1 | 25.4 |
| Heart problems and treatment | 7 | 68 | 78.2 | 17.6 | 1.8 | 35.8 |
| Treatment II | 3 | 68 | 95.3 | 9.4 | 0.0 | 80.0 |
| Perceived physical appearance | 3 | 68 | 93.4 | 12.8 | 2.2 | 75.0 |
| Treatment anxiety | 4 | 68 | 67.6 | 28.8 | 4.6 | 31.2 |
| Cognitive problems | 5 | 68 | 68.4 | 19.4 | 17.3 | 34.6 |
| Communication | 3 | 68 | 71.4 | 27.0 | 10.0 | 37.1 |
| **Parent Proxy-Report, Mother (5-7 years)** |  |  |  |  |  |  |
| Total Score | 25 | 68 | 74.5 | 14.8 | 13.5 | 24.5 |
| Heart problems and treatment | 7 | 68 | 80.9 | 15.7 | 2.5 | 36.7 |
| Treatment II | 3 | 68 | 97.7 | 7.5 | 0.0 | 90.9 |
| Perceived physical appearance | 3 | 68 | 86.9 | 16.5 | 3.5 | 58.3 |
| Treatment anxiety | 4 | 68 | 68.3 | 27.1 | 6.3 | 28.4 |
| Cognitive problems | 5 | 68 | 65.0 | 22.4 | 18.1 | 29.8 |
| Communication | 3 | 68 | 66.5 | 28.4 | 12.7 | 35.4 |
| **Parent Proxy-Report, Father (8-12;13-18 years)** |  |  |  |  |  |  |
| Total Score | 27 | 295 | 78.4 | 14.1 | 9.6 | 28.2 |
| Heart problems and treatment | 7 | 295 | 81.3 | 15.7 | 3.2 | 37.5 |
| Treatment II | 5 | 295 | 95.6 | 6.8 | 0.0 | 62.7 |
| Perceived physical appearance | 3 | 295 | 79.8 | 24.2 | 6.6 | 47.7 |
| Treatment anxiety | 4 | 295 | 70.8 | 25.3 | 3.4 | 30.6 |
| Cognitive problems | 5 | 295 | 72.3 | 21.8 | 8.6 | 29.1 |
| Communication | 3 | 295 | 80.2 | 23.3 | 5.3 | 44.0 |
| **Parent Proxy-Report, Mother (8-12;13-18 years)** |  |  |  |  |  |  |
| Total Score | 27 | 295 | 75.0 | 15.1 | 9.6 | 28.2 |
| Heart problems and treatment | 7 | 295 | 78.8 | 14.8 | 2.9 | 35.1 |
| Treatment II | 5 | 295 | 93.4 | 9.2 | 2.0 | 59.7 |
| Perceived Physical appearance | 3 | 295 | 75.1 | 25.8 | 6.3 | 40.0 |
| Treatment anxiety | 4 | 295 | 66.7 | 27.9 | 6.1 | 27.2 |
| Cognitive problems | 5 | 295 | 68.5 | 24.6 | 10.8 | 27.8 |
| Communication | 3 | 295 | 76.3 | 25.6 | 5.2 | 41.8 |

**Table 7. PedsQL 3.0 Cardiac Module Scores, Reliability and percent Floor and Ceiling Effects for Child Self-Report and Parent Proxy-Report**

| **Generic Module Scales** | **Number of items** | **n** | **Mean** | **SD** | **% Floor** | **% Ceiling** |
| --- | --- | --- | --- | --- | --- | --- |
| **Child Self-Report (5-7 years)** |  |  |  |  |  |  |
| Total Score | 23 | 68 | 80.7 | 12.3 | 20.7 | 40.2 |
| Physical Functioning | 8 | 68 | 80.7 | 16.3 | 11.7 | 46.9 |
| Emotional Functioning | 5 | 68 | 72.5 | 18.5 | 10.5 | 63.9 |
| Social Functioning | 5 | 68 | 86.7 | 17.0 | 8.1 | 55.8 |
| School Functioning | 5 | 68 | 82.4 | 18.4 | 12.2 | 57.0 |
| **Child Self-Report (8-12;13-18 years)** |  |  |  |  |  |  |
| Total Score | 23 | 295 | 79.7 | 12.1 | 20.7 | 40.2 |
| Physical Functioning | 8 | 295 | 80.3 | 15.2 | 11.5 | 48.5 |
| Emotional Functioning | 5 | 295 | 72.6 | 16.7 | 11.3 | 46.2 |
| Social Functioning | 5 | 295 | 87.7 | 15.1 | 4.7 | 61.3 |
| School Functioning | 5 | 295 | 77.4 | 18.6 | 6.2 | 54.4 |
| **Parent Proxy-Report, Father (2-4years)** |  |  |  |  |  |  |
| Total Score | 21 | 37 | 81.7 | 14.3 | 10.7 | 28.5 |
| Physical Functioning | 8 | 37 | 82.7 | 17.2 | 4.9 | 39.3 |
| Emotional Functioning | 5 | 37 | 73.9 | 21.4 | 7.2 | 30.9 |
| Social Functioning | 5 | 37 | 88.9 | 17.3 | 5.4 | 56.7 |
| School Functioning | 3 | 37 | 79.3 | 22.3 | 6.9 | 46.5 |
| **Parent Proxy-Report, Mother (2-4years)** |  |  |  |  |  |  |
| Total Score | 21 | 37 | 78.3 | 16.8 | 8.6 | 29.3 |
| Physical Functioning | 8 | 37 | 78.3 | 16.8 | 5.1 | 40.2 |
| Emotional Functioning | 5 | 37 | 69.4 | 19.3 | 5.6 | 27.3 |
| Social Functioning | 5 | 37 | 87.7 | 19.9 | 3.3 | 55.9 |
| School Functioning | 3 | 37 | 74.4 | 23.1 | 3.5 | 42.8 |
| **Child Proxy-Report, Father (5-7 years)** |  |  |  |  |  |  |
| Total Score | 23 | 68 | 83.3 | 11.1 | 4.8 | 31.4 |
| Physical Functioning | 8 | 68 | 83.3 | 15.7 | 2.4 | 41.2 |
| Emotional Functioning | 5 | 68 | 78.9 | 15.1 | 1.2 | 36.1 |
| Social Functioning | 5 | 68 | 90.3 | 2.9 | 12.0 | 52.2 |
| School Functioning | 5 | 68 | 82.2 | 18.2 | 2.5 | 40.0 |
| **Child Proxy-Report, Mother (5-7 years)** |  |  |  |  |  |  |
| Total Score | 23 | 68 | 80.7 | 12.3 | 20.7 | 40.2 |
| Physical Functioning | 8 | 68 | 80.7 | 16.3 | 11.7 | 46.9 |
| Emotional Functioning | 5 | 68 | 75.7 | 16.1 | 0.68 | 35.4 |
| Social Functioning | 5 | 68 | 86.7 | 17.0 | 8.1 | 55.8 |
| School Functioning | 5 | 68 | 82.4 | 18.4 | 12.2 | 57.0 |
| **Child Proxy-Report, Mother (8-12;13-18 years)** |  |  |  |  |  |  |
| Total Score | 23 | 68 | 80.7 | 12.3 | 20.7 | 40.2 |
| Physical Functioning | 8 | 68 | 80.7 | 16.3 | 11.7 | 46.9 |
| Emotional Functioning | 5 | 68 | 72.7 | 18.3 | 10.5 | 63.9 |
| Social Functioning | 5 | 68 | 86.7 | 17.0 | 8.1 | 55.8 |
| School Functioning | 5 | 68 | 82.4 | 18.4 | 12.2 | 57.0 |
| **Child Proxy-Report, Father (8-12;13-18 years)** |  |  |  |  |  |  |
| Total Score | 23 | 68 | 80.7 | 12.3 | 20.7 | 40.2 |
| Physical Functioning | 8 | 68 | 80.7 | 16.3 | 11.7 | 46.9 |
| Emotional Functioning | 5 | 68 | 75.5 | 19.6 | 10.5 | 63.9 |
| Social Functioning | 5 | 68 | 86.7 | 17.0 | 8.1 | 55.8 |
| School Functioning | 5 | 68 | 82.4 | 18.4 | 12.2 | 57.0 |

**Table 8. PedsQL 4.0 Generic Module Scores, Reliability and percent Floor and Ceiling Effects for Child Self-Report and Parent Proxy-Report**
